# Simple, Rapid and Cost-Effective Method for High Quality Nucleic Acids Extraction from Different Strains of *Botryococcus braunii*

**DOI:** 10.1371/journal.pone.0037770

**Published:** 2012-05-25

**Authors:** Byung-Hyuk Kim, Rishiram Ramanan, Dae-Hyun Cho, Gang-Guk Choi, Hyun-Joon La, Chi-Yong Ahn, Hee-Mock Oh, Hee-Sik Kim

**Affiliations:** 1 Environmental Biotechnology Research Center, KRIBB, Daejeon, Republic of Korea; 2 Green Chemistry and Environmental Biotechnology, University of Science and Technology, Daejeon, Republic of Korea; Lawrence Berkeley National Laboratory, United States of America

## Abstract

This study deals with an effective nucleic acids extraction method from various strains of *Botryococcus braunii* which possesses an extensive extracellular matrix. A method combining freeze/thaw and bead-beating with heterogeneous diameter of silica/zirconia beads was optimized to isolate DNA and RNA from microalgae, especially from *B. braunii*. Eukaryotic Microalgal Nucleic Acids Extraction (EMNE) method developed in this study showed at least 300 times higher DNA yield in all strains of *B. braunii* with high integrity and 50 times reduced working volume compared to commercially available DNA extraction kits. High quality RNA was also extracted using this method and more than two times the yield compared to existing methods. Real-time experiments confirmed the quality and quantity of the input DNA and RNA extracted using EMNE method. The method was also applied to other eukaryotic microalgae, such as diatoms, *Chlamydomonas* sp., *Chlorella* sp., and *Scenedesmus* sp. resulting in higher efficiencies. Cost-effectiveness analysis of DNA extraction by various methods revealed that EMNE method was superior to commercial kits and other reported methods by >15%. This method would immensely contribute to area of microalgal genomics.

## Introduction

Increasingly, microalgae are gaining interest among molecular biologists and biotechnologists for its ability to produce value-added products. Microalgae are perceived to be sunlight-driven cell factories that convert carbon dioxide to potential biofuels, food supplements, feeds and other high-value products [1]. *Botryococcus braunii* is a freshwater colonial green microalgae that produces hydrocarbons up to 86% of its dry weight [2]. Its ability to produce relatively high quantity of hydrocarbons has resulted in the organism being proposed as a future renewable resource [3], [4]. Recently, algal biofuels has been one of the most widely researched areas [5]. Different research strategies including nutrient regime, novel photobioreactor design and genetic manipulation of microalgae are being carried out in order to achieve higher lipid productivity in microalgal strains. However, the production of lipids from microalgae has remained unfeasible commercially due to a combination of factors. Genetic engineering of microalgae has been proposed to be one of the most promising alternative for commercializing microalgal lipid production through strain improvement [6], [7].

Meanwhile, for the characterization of individual members of microalgae or for genetic engineering, cloning and sequence analysis of DNA or cDNA are employed. The initial steps of all these procedures entail direct extraction of DNA or RNA. In addition, other techniques, such as blotting and polymerase chain reaction (PCR), also need quick isolation of intact nucleic acids without any cross-contamination. The various methods that have already been proposed to extract and purify the nucleic acids from cells can be classified according to the system chosen to break the cells outer structures: bead beating, enzymatic cell wall lysis, and cell permeabilization using chaotropic agents [8], [9]. However, all these methods are either too time consuming or expensive to use for nucleic acids isolation from microalgae. Therefore, harsh conditions are required to disrupt the highly resistant cell wall of microalgae [10]. In addition, traditional methods for nucleic acid extraction from microalgae require large amounts of cells due to low yields. However, many microalgae including *B. braunii* have a low growth rate resulting in low cell yields even under controlled laboratory conditions.

**Figure 1:**
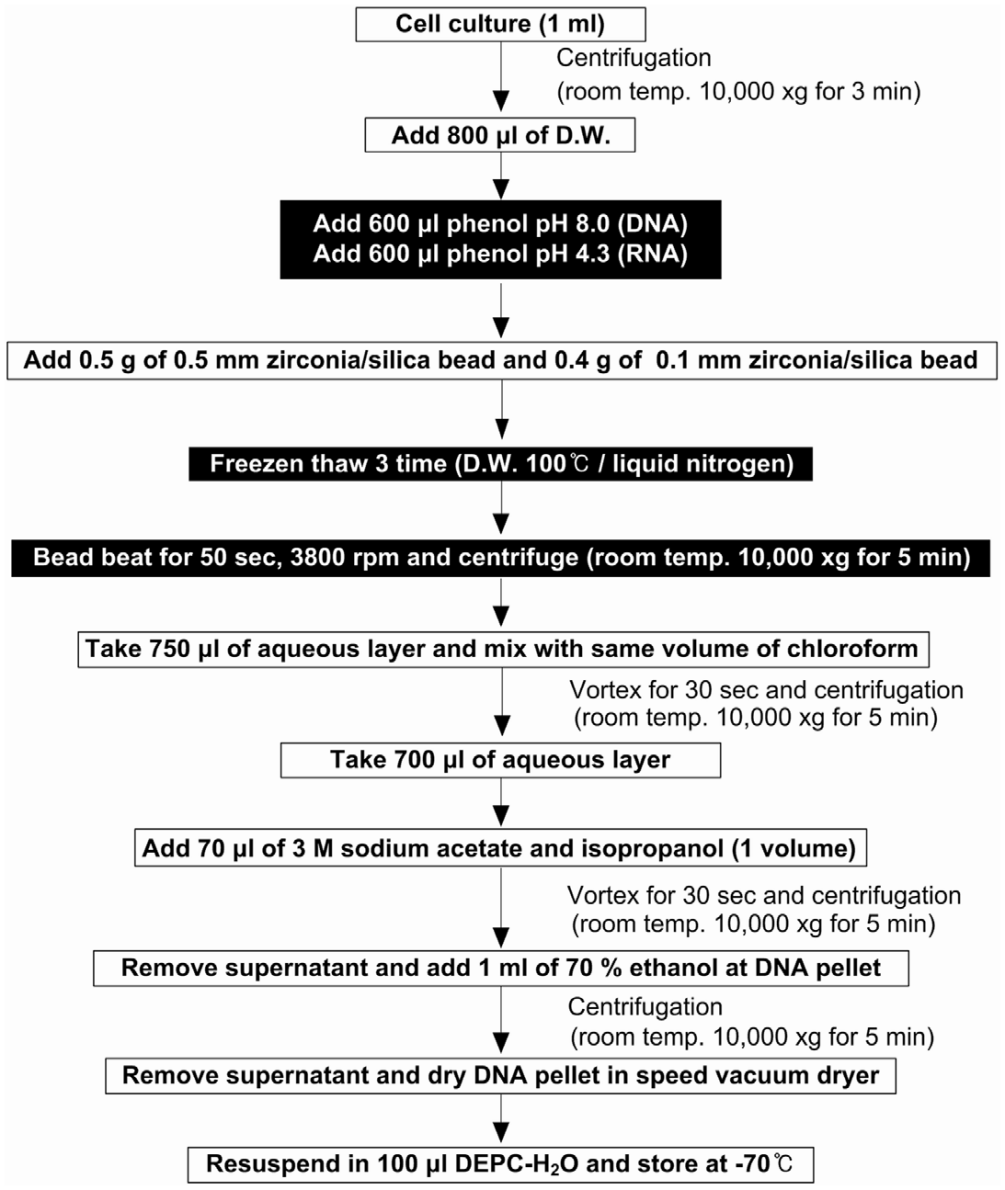
Summary of nucleic acids extraction from eukaryotic microalgae using EMNE method.

Many eukaryotic microalgae have highly resistant cell walls containing lignin-like components and/or sporopollenin [11]. It is widely known that the thick hydrocarbon matrix surrounding individual cells forms an outer cell wall of *B. braunii* [12]. They are chemically very stable and usually well preserved in environments. Therefore, genomic investigations for the physiology, function and phylogenetic distribution of eukaryotic microalgae have been limited. Accordingly, this study presents a novel and efficient method for the preparation of nucleic acids from eukaryotic microalgae with highly resistant cell walls and thick outer membranes. The eukaryotic microalgal nucleic acids extraction (EMNE) method is a combination of modified Bead-Phenol-Chloroform (BPC) method [13] and modified freeze/thaw (F&T) method [14]. The EMNE method involves the use of freeze/thaw, zirconia/silica beads, bead-beating, and phenol to break the rigid cell walls and to extract DNA.

## Materials and Methods

### Strains and Cultivation

Eleven eukaryotic microalgal strains (Green algae; *B. braunii* CCALA 776, *B. braunii* CCALA 777, *B. braunii* CCALA 778, *B. braunii* CCALA 779, *B. braunii* UTEX 572, *B. braunii* NIES 836, *Chlamydomonas reinhardtii* KCTC AG 20446, *Chlorella* sp. KCTC AG 10032, and *Scenedesmus* sp. KCTC AG 20831. Diatom; *Phaeodactylum tricornutu* KCTC AG 30124, *Pleurochrysis carterae* KCTC AG 40012) were used in this study. The axenic cultures of these strains were obtained from Biological Resource Centre (BRC), KRIBB, Daejeon, South Korea.

Green algae were cultured in 500-ml Erlenmeyer flasks in BG11 medium at 30°C for 3 weeks with a light intensity of 50 µEm^−2^ s^−1^. Diatoms were cultured in 1% f/2 medium at 25°C for 1 month. The dry cell weight of eleven eukaryotic microalgal strains were *B. braunii* CCALA 776; 1.17 mg/ml, *B. braunii* CCALA 777; 1.48 mg/ml, *B. braunii* CCALA 778; 1.1 mg/ml, *B. braunii* CCALA 779; 1.38 mg/ml, *B. braunii* UTEX 572; 2.3 mg/ml, *B. braunii* NIES 836; 2.12 mg/ml, *Chlamydomonas reinhardtii* KCTC AG 20446; 1.62 mg/ml, *Chlorella* sp. KCTC AG 10032; 2.49 mg/ml, and *Scenedesmus* sp. KCTC AG 20831; 5.5 mg/ml, *Phaeodactylum tricornutu* KCTC AG 30124; 1.71 mg/ml, *Pleurochrysis carterae* KCTC AG 40012; 2.57 mg/ml, respectively.

**Figure 2:**
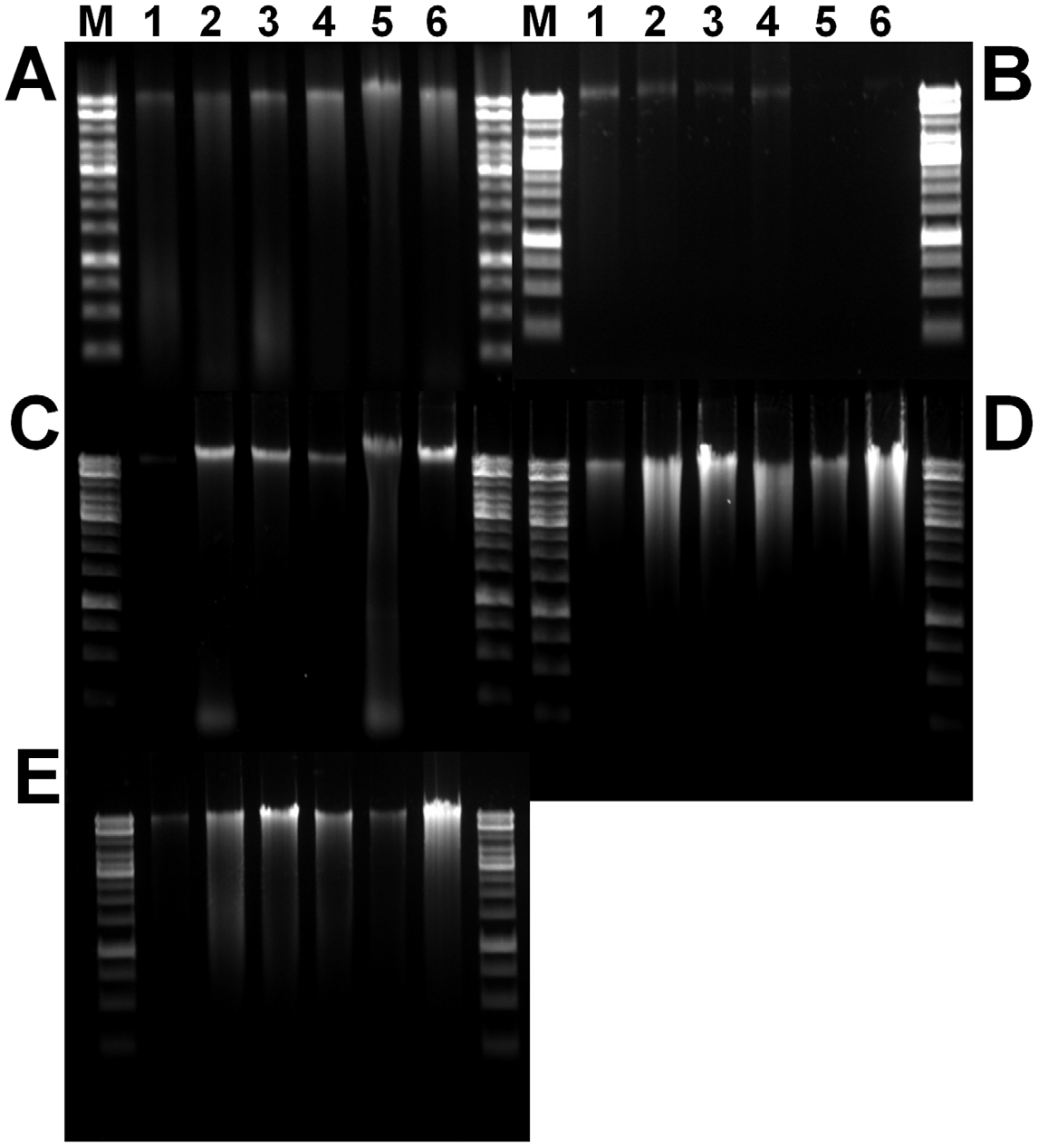
Gel electrophoresis of total DNA extracted from *B. braunii* and other eukaryotic microalgae. DNA samples were electrophoresed on a 1.6% agarose gel in 0.5× TAE buffer. (a) commercial method 1 (P Kit); (b) commercials method 2 (Q Kit), (c) F&T method; (d) BPC method; (e) EMNE method. Lane M, 1 Kb ladder; lane 1, *B. braunii* CCALA 776; lane 2, *B. braunii* CCALA 777; lane 3, *B. braunii* CCALA 778; lane 4, *B. braunii* CCALA 779; lane 5, *B. braunii* UTEX 572; lane 6, *B. braunii* NIES 836.

**Figure 3:**
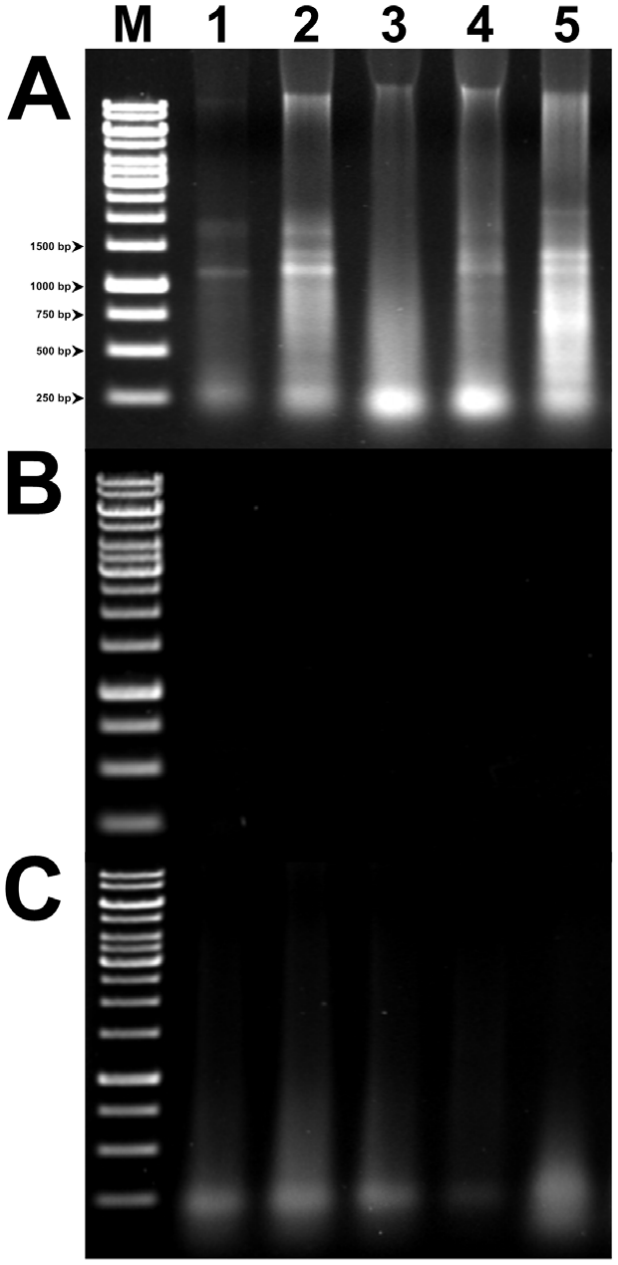
Gel electrophoresis of total RNA extracted from *Botryococcus* sp by EMNE (A), F&T (B), and BPC method (C). RNA samples were electrophoresed on a 1.6% agarose gel in 0.5× TAE buffer. Lane M, 1 Kb ladder; lane 1, *Botryococcus braunii* CCALA 776; lane 2, *B. braunii* CCALA 777; lane 3, *B. braunii* CCALA 778; lane 4, *B. braunii* CCALA 779; lane 5, *B. braunii* UTEX 572.

### Nucleic Acids Extraction by EMNE method

The 1 ml cultured cells were harvested by centrifugation at 10,000×g for 3 min, then the cell pellets were resuspended in 800 µl of deionized water and mixed with 600 µl of saturated phenol (pH 8.0) for DNA extraction and same volume of saturated phenol (pH 4.3) for RNA extraction. Rest of the steps remained the same for either nucleic acids and all procedures were carried out at room temperature, unless otherwise stated. Zirconia/silica beads (0.5 g, 0.5 mm in diameter/0.4 g, 0.1 mm in diameter; Biospec, Oklahoma, USA) were added to the cell suspensions. Then, three cycles of freezing in a liquid nitrogen bath and thawing in a 100°C water bath were conducted to partially break the cells. After the freeze/thaw cycles, bead beating for 50 sec at 3,800 rpm using a Mini beadbeater (Biospec) was performed to completely break the cells and release the nucleic acid from the samples. The samples were then centrifuged at 10,000×g for 5 min and the supernatants (750 µl) transferred to a fresh 1.5 ml-microtube. The transferred samples were then mixed with same volume of chloroform and centrifuged at 10,000 X g for 5 min. Thereafter, the aqueous layer (700 µl) was transferred to another fresh 1.5 ml microtube and mixed with same volume of isopropanol and 3 M sodium acetate (pH. 8.0, 1/10^th^ volume). The nucleic acid pellet obtained by centrifugation at 10,000×g for 5 min was washed with ice-cold 70% ethanol and dried in a centrifugal vacuum dryer (Speed Vac SWT120, Savant, USA). Finally, the dried nucleic acid samples were resuspended in 100 µl H_2_O and analyzed on a 1.6% agarose gel. The efficiency and purity of the nucleic acids was quantified using Nanodrop ND-1000 (Thermo Scientific, DE, USA) at 260 nm/280 nm and 260 nm/230 nm. The Nucleic acids isolation procedure by EMNE method has been summarized in Figure 1. EMNE method was compared with BPC method [13], F&T method [14] and commercial kits, such as DNeasy® Plant mini kit [Q Kit] (Qiagen, CA, USA) and Wizard® Genomic DNA purification kit [P Kit] (Promega, WI, USA). A commercial bead beating kit was also tested for its efficiency (MPBio FastDNA Spin Kit, MP Bio, USA). About 3 ml of algal culture was used for DNA extraction using BPC and F&T methods whereas 50 ml of culture was used to extract DNA using commercial DNA extraction kits. All the experimental sets were performed in triplicates.

**Table 1:** Comparison of quality and quantity of DNA samples extracted from B. *braunii* using different methods.

Methods	Strain	A260/280^a^	A260/230	DNA Conc. (ng/ul)^b^	Total DNA (µg)	Dry Cell Weight^c^	DNA Yield (µg/mg)^d^
P kit	*B.braunii* CCALA 776	2.01	0.46	77.90	7.790	58.50	0.133
	*B. braunii* CCALA 777	2.01	0.55	76.90	7.690	74.00	0.104
	*B. braunii* CCALA 778	1.29	0.58	59.06	5.906	55.00	0.107
	*B. braunii* CCALA 779	1.84	0.51	36.85	3.685	69.00	0.053
	*B. braunii* UTEX 572	1.71	0.49	27.40	2.740	115.00	0.024
	*B. braunii* NIES 836	1.80	0.48	42.95	4.295	106.00	0.041
Q kit	*B. braunii* CCALA 776	1.80	0.23	38.75	3.875	58.50	0.066
	*B. braunii* CCALA 777	1.71	0.28	37.95	3.795	74.00	0.051
	*B. braunii* CCALA 778	1.72	0.33	44.90	4.490	55.00	0.082
	*B.braunii* CCALA 779	1.69	0.39	49.10	4.910	69.00	0.071
	*B. braunii* UTEX 572	1.67	0.34	45.65	4.565	115.00	0.040
	*B. braunii* NIES 836	1.64	0.42	46.00	4.600	106.00	0.043
F&T	*B. braunii* CCALA 776	2.25	1.89	24.95	2.495	3.51	0.711
	*B. braunii* CCALA 777	2.09	2.04	87.45	8.745	4.44	1.970
	*B.braunii* CCALA 778	1.97	1.95	57.35	5.735	3.30	1.738
	*B. braunii* CCALA 779	2.02	2.10	53.35	5.335	4.14	1.289
	*B. braunii* UTEX 572	2.09	2.03	98.05	9.805	6.90	1.421
	*B. braunii* NIES 836	2.05	1.91	40.25	4.025	6.36	0.633
BPC	*B. braunii* CCALA 776	2.01	1.95	114.10	11.410	3.51	3.251
	*B. braunii* CCALA 777	1.92	2.04	298.60	29.860	4.44	6.725
	*B. braunii* CCALA 778	1.96	1.95	270.10	27.010	3.30	8.185
	*B. braunii* CCALA 779	1.96	2.10	189.65	18.965	4.14	4.581
	*B. braunii* UTEX 572	2.07	2.03	199.75	19.975	6.90	2.895
	*B. braunii* NIES 836	1.67	1.91	413.20	41.320	6.36	6.497
EMNE	*B.braunii* CCALA 776	1.72	1.94	117.75	11.775	1.17	10.064
	*B. braunii* CCALA 777	1.83	2.04	511.45	51.145	1.48	34.557
	*B. braunii* CCALA 778	1.85	1.98	426.20	42.620	1.10	38.745
	*B. braunii* CCALA 779	1.95	2.17	287.95	28.795	1.38	20.866
	*B. braunii* UTEX 572	2.05	2.19	210.90	21.090	2.30	9.170
	*B. braunii* NIES 836	1.89	2.09	758.65	75.865	2.12	35.785

a The DNA purity was estimated by calculating the ratio between the absorbance at 260 nm and 280 nm.

b The DNA concentration was quantified using a spectrophotometer at 260 nm.

c The cultured cells were harvested from culture of 50 ml (commercial kit), 3 ml (BPC & F&T methods), and 1 ml (EMNE method for *B. braunii*), respectively.

d The DNA yield was estimated by calculating the DNA concentration between the dry cell weight and total DNA.

CCALA, Culture Collection of Autotrophic Organisms; NIES, National Institute for Environmental Studies; UTEX, the Culture Collection of Algae at the University of Texas.

**Table 2:** Comparison of yield and purity of RNA samples prepared by EMNE, F&T, and BPC method.

Methods	Strain	260/280^a^	A260/230	RNA Conc. (ng/ul)^b^	Total RNA (ug)	Dry Cell Weight^c^	RNA Yield (µg/mg)
F&T	*B. braunii* CCALA 776	1.76	1.80	8.0	0.8	3.57	0.225
	*B. braunii* CCALA 777	1.47	1.82	9.2	0.9	4.74	0.195
	*B. braunii* CCALA 778	1.60	2.01	8.2	0.8	5.61	0.147
	*B. braunii* CCALA 779	1.64	1.85	9.0	0.9	3.51	0.258
	*B. braunii* UTEX 572	1.66	1.95	6.6	0.7	7.26	0.091
BPC	*B. braunii* CCALA 776	1.89	1.98	81.4	8.1	3.57	2.281
	*B. braunii* CCALA 777	1.88	1.82	93.8	9.4	4.74	1.978
	*B. braunii* CCALA 778	1.93	1.93	97.4	9.7	5.61	1.735
	*B. braunii* CCALA 779	1.99	2.04	30.7	3.1	3.51	0.875
	*B. braunii* UTEX 572	1.96	1.85	112.0	11.2	7.26	1.543
EMNE	*B. braunii* CCALA 776	1.87	1.95	158.0	15.8	1.19	13.281
	*B. braunii* CCALA 777	1.94	1.97	224.4	22.4	1.58	14.205
	*B. braunii* CCALA 778	1.72	1.89	290.0	29.0	1.87	15.506
	*B. braunii* CCALA 779	1.86	2.09	170.1	17.0	1.17	14.534
	*B. braunii* UTEX 572	1.89	2.02	337.6	33.8	2.42	13.949

a The RNA purity was estimated by calculating the ratio between the absorbance at 260 nm and 280 nm.

b The RNA concentration was quantified using a spectrophotometer at 260 nm.

c The cultured cells were harvested from culture of 3 ml (BPC & F&T methods) and 1 ml (EMNE method for *B. braunii*), respectively.

CCALA, Culture Collection of Autotrophic Organisms; UTEX, the Culture Collection of Algae at the University of Texas at Austin.

### Real Time PCR for DNA Integrity Analysis

The 18 S rRNA gene specific to *Botryococcus* sp. was amplified using primers [15], Chlo-165F (5′-CGA CTT CTG GAA GGG ACG TA-3′) and Chlo-595R (5′-ATT GGA GCT GGA ATT ACC GC-3′), resulting in a 431 bp PCR product. Moreover, another size of the 18 S rRNA gene was amplified using primer Bo-213F (5′-GTT CAC GGG TGA CGG AGA AT-3′) and Bo-213R (5′-CAC CAG ACT TGC CCT CCA AT-3′), Bo-527F (5′-TGC CTG ACT CTT GCT GAT TCA T -3′) and Bo-527R (5′-ACT CAA AGT AAC CAC GCC GACT -3′), resulting in a 213 bp and 527 bp PCR product, respectively. The amplification of *Botryococcus* sp. specific *rBcL* gene was performed using rBcL B-2F (5′-GGT GGT GAT CAC TTG CAT TC-3′) and rBcL B-2R (5′-TCA CGA CCT TCG TTA CGA GCT T-3′) resulting in a 343 bp PCR product. The 18 S rRNA genes and *rBcL* gene sequences were deposited in the NCBI database under GenBank accession numbers JQ941954 and JQ941955.

PCR was performed in 30 µl reactions containing 2× PCR premix (EF) (Solgent, Korea), 0.5 µM concentrations of each primer, and 50 ng of DNA. The conditions used for PCR amplification were as follows: initial denaturation at 94°C for 5 min and then 30 cycles of (denaturation 94°C for 1 min, primer annealing at 63°C for 1 min, and chain extension for 1 min at 72°C), followed by a final extension at 72°C for 5 min. All products were cloned into a T-Blunt vector (Solgent, Korea) according to the manufacturer’s protocol to create artificial standard clones for real-time PCR analysis. Plasmid DNA was extracted from overnight *Escherichia coli* DH5α cultures using the LaboPass Mini Kit (CosmoGen, Korea) and sequenced with M13F and M13R (T-Blunt Vector Systems manual). Nearly full-length 18S rRNA gene sequences were assembled with SeqMan II (DNAstar) using the best match sequence of each operational taxonomic unit (OTU) on Genbank (www.ncbi.nlm.nih.gov/BLAST) as a template.

Quantitative real-time PCR was performed using Chromo4 (Bio-Rad, Hercules, CA) thermalcycler and iTaq™ SYBR® Green Supermix with ROX (Bio-Rad, CA, USA) to compare the PCR amplification efficiency of DNA obtained by different methods. Each 20 µl reaction contained 10 µl of SYBR Green Supermix with ROX, 7 µl sterile water, 1 µl of DNA template, and each forward and reverse primers at 1 µl (10 pmole/µl). For *Botryococcus* sp. 18 S rRNA gene qPCR, 18 S rRNA gene specific primers were used, and thermocycling program was as follows: initial denaturation for 3 min at 95°C, 45 cycles of denaturation at 95°C for 30 sec, annealing at 68°C, 63°C and 69°C for 30 sec and extension at 72°C for 30 sec; and a final melting curve analysis from 65°C to 95°C, measuring fluorescence every 0.2°C, respectively. For *rBcL* gene qPCR, similar conditions were used, except the primers used were rBcL B-2F and rBcL B-2R the annealing temperature were 67°C, respectively.

Calibration was performed with serial dilutions of known quantity of one of *Botryococcus* sp. 18 S rRNA gene and *rBcL* gene-containing standard clones. DNA concentrations were determined with NanoDrop ND-1000 (Thermo Scientific, DE, USA). Differences between 18 S rRNA gene copies/µl and *rBcL* gene copies/µl at different sampling time points were compared with a one-tailed Student *t* test.

### cDNA Synthesis and qPCR

Reverse transcription (RT) and qPCR analysis for genes (18 S rRNA genes and *rbcL*) of *B. braunii* were carried out according to commercial cDNA Synthesis Kit (iScript cDNA Synthesis Kit, Bio-Rad, USA). For RT-qPCR analysis of *B. braunii*, DNA was removed by incubating with RNase-free DNase (Recombinant DNase I. Takara, Japan) at 37°C for 30 min. The RT reaction mixtures were incubated at 42°C for 30 min. qPCR was performed on 20 µl of the RT reaction under the same conditions described above. RNA was used as negative control to check for cross-contamination.

**Figure 4:**
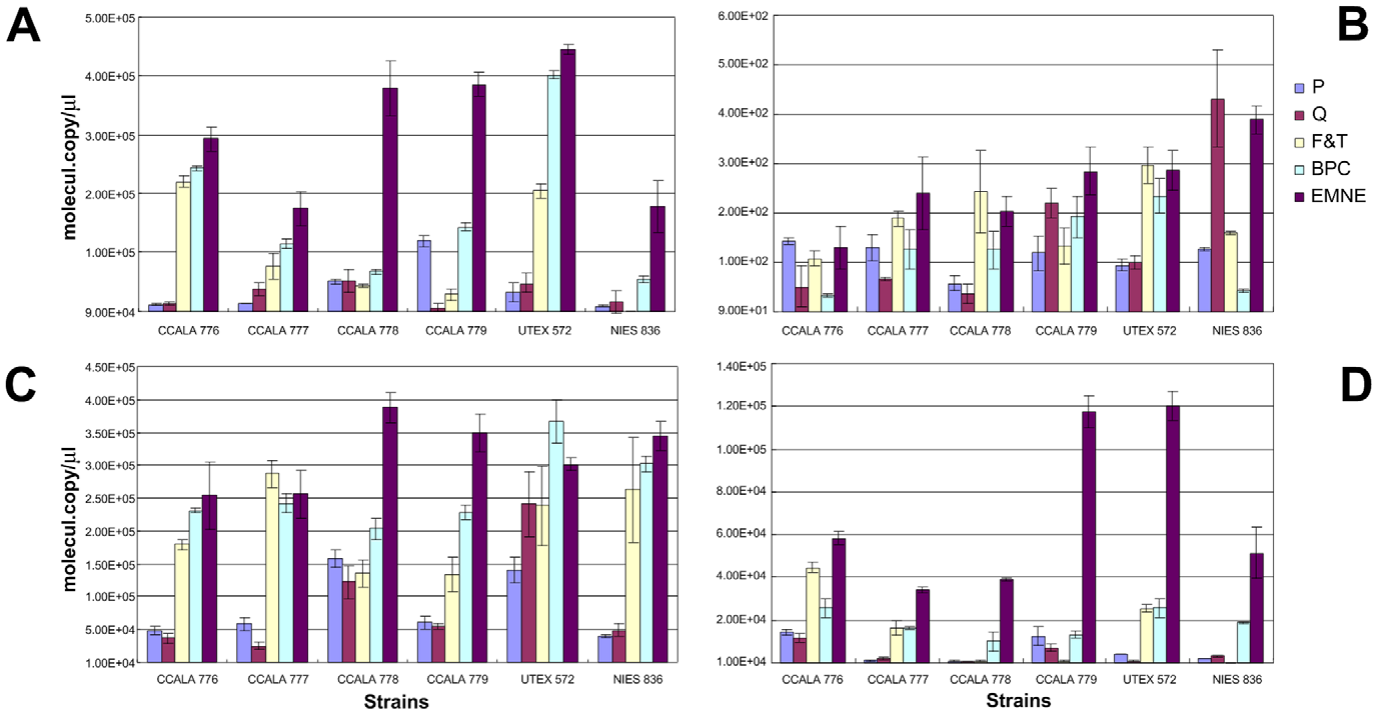
qPCR of *B. braunii* NIES 836 18 S rRNA gene of 213 bp (A), *rBcL* gene of 343 bp (B), 18 S rRNA gene of 431 bp (C), and 18 S rRNA gene of 527 bp (D) from DNA of *B. braunii* sp. Closed square; P kit, open square; Q kit, closed diamond; F&T method, open diamond; BPC method, closed triangle; EMNE method.

**Figure 5:**
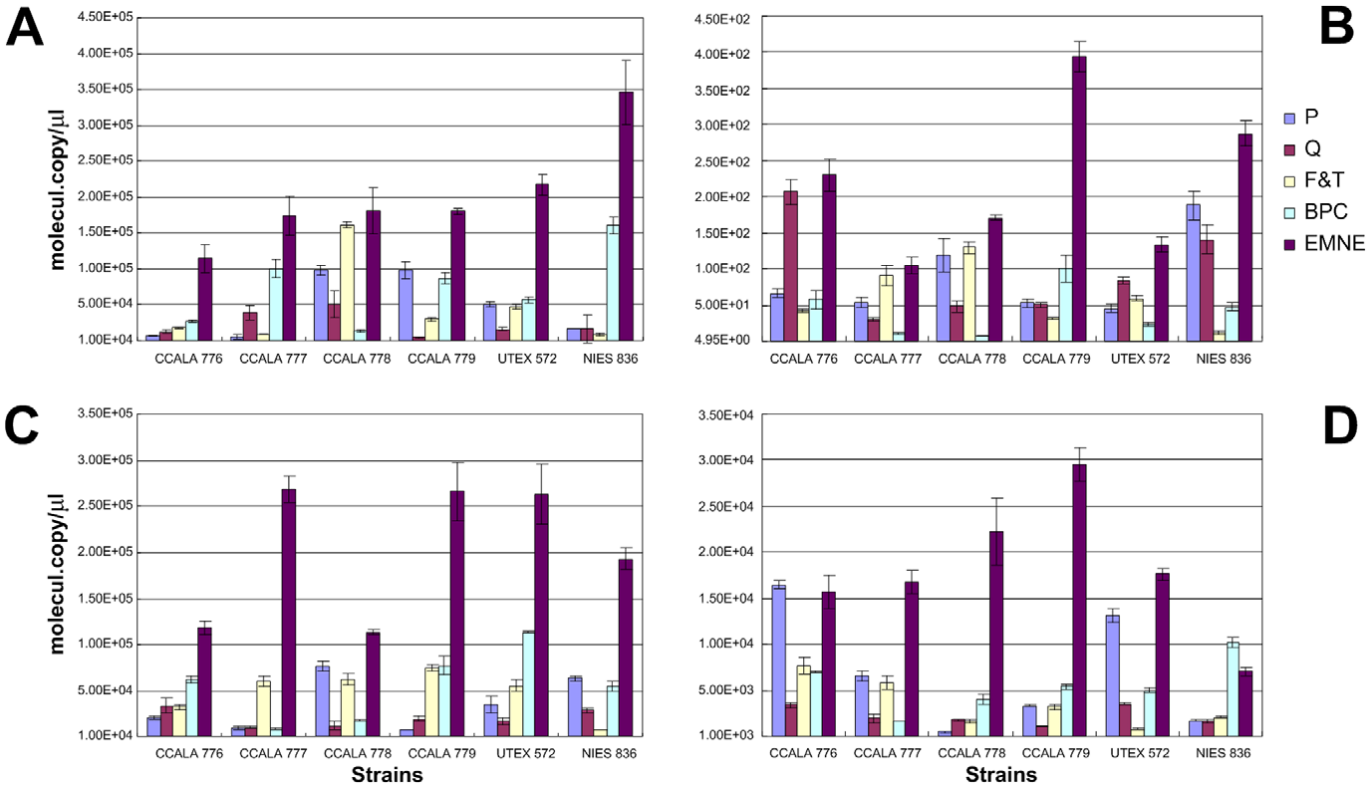
RT-qPCR of *B. braunii* NIES 836 18 S rRNA gene of 213 bp (A), *rBcL* gene of 343 bp (B), 18 S rRNA gene of 431 bp (C), and 18 S rRNA gene of 527 bp (D). Closed square; P kit, open square; Q kit, closed diamond; F&T method, open diamond; BPC method, closed triangle; EMNE method.

### Cost-Effectiveness Analysis

Direct measurements were made in enumerating the cost of inputs from the manufacturer’s pricing, including equipment costs, and all costs were converted into USD. The effectiveness of each method was averaged from the experimental results for each parameter. Relative performance index was calculated depending upon values of cost and effectiveness for each method with a best score of 10 and worst of 0 [16]. For e.g. effectiveness of each method in terms of DNA quality was determined by calculating the average 260/280 nm among all strains for each method, followed by assigning a baseline score of 10 for the best performing method. Relative scoring from the baseline score of 10 was assigned for each method by calculating relative percentage of performance. For arriving at effectiveness of each method in terms of DNA integrity, the average value of Co-variance of copy number values of *rBcL* gene among different strains was used. For arriving at effectiveness of each method in terms of efficiency for downstream applications, the mean of copy number values for different sizes of 18 S rRNA gene was used as copy number is directly dependent on the quality and quantity of the input DNA/cDNA sample. Total score reflects the cost-effectiveness of each method.

### Statistical Analysis

Statistical analysis of qPCR data was performed to ascertain the variance among methods and strains using One Way Analysis of Variance (ANOVA). Co-variance was calculated as a ratio of standard deviation of copy number value obtained for each method and mean of copy number values.

**Table 3:** Cost Effectiveness Analysis of different methods used for DNA extraction.

Cost-Effectiveness Analysis - Parameters	BaselineCost-EffectivenessScore	P	Q	F&T	BPC	EMNE
DNA concentration	10	6.62	3.29	2.12	9.69	10.00
DNA yield	10	0.03	0.02	0.52	2.15	10.00
DNA quality (260/280)	10	8.55	8.20	10.00	9.29	9.05
DNA integrity	10	10.00	3.17	4.93	5.77	8.02
Efficiency for downstream applications	10	1.86	1.73	4.55	6.50	10.00
Time & Labour Requirement	10	7.50	7.50	10.00	10.00	10.00
Capital cost on materials	10	7.73	10.00	4.03	3.29	3.287
Capital cost on equipments	10	0.67	0.67	10.00	0.0113	0.0113
Recurring cost	10	7.73	10.00	4.03	3.29	3.287
Cost per sample	10	2.53	1.64	10.00	8.45	8.43
**Total Score**	**100**	**53.22**	**46.23**	**60.17**	**58.45**	**72.09**

Baseline scores and relative scores for each parameter for different methods have been depicted.

## Results

For genetic studies in microalgae, a fast, simple, and reliable nucleic acids extraction method is a prerequisite. This study deals with an effective nucleic acids extraction method from *B. braunii* and other eukaryotic algae (Figure 1). Total DNA extracted from *B. braunii* strains by different methods such as EMNE, BPC, F&T, and commercial kits were analyzed by electrophoresis on an agarose gel (Figure 2). Total RNA isolated from *B. braunii* strains by EMNE was also analyzed by agarose gel electrophoresis (Figure 3). DNA extracted from commercial kits revealed a weak band in almost all the samples. Moreover, DNA could not be extracted from UTEX 572 and NIES 836 strains using commercial kits (Figure 2A and 2B). F&T method resulted in a very faint DNA band for CCALA 776 strain (Figure 2C). DNA extraction using BPC and EMNE for all *B. braunii* strains yielded a strong band as evidenced in Figure 2D and 2E. RNA extraction also yielded high intensity bands for all strains by EMNE (Figure 3A), whereas faint bands were evidenced for RNA extraction using BPC method (Figure 3C). However, agarose gel electrophoresis did not reveal any band while using F&T method (Figure 3B). Additionally, DNA extracted from *B. braunii* using the commercial bead beating kit showed very low DNA yield, which could not be detected by agarose gel electrophoresis (data not shown).

### Nucleic Acids Yield

The average concentration of extracted DNA was 43.725 ng/µl (Q Kit), 53.509 ng/µl (P Kit), 60.233 ng/µl (BPC), 247.567 ng/µl (F&T) and 385.48 ng/µl (EMNE). A comparison of the DNA yield using similar initial culture mass revealed that the average DNA yield of EMNE method was 24.865 µg/mg, while the average DNA yield of commercial kits, BPC and F&T were 0.059 µg/mg (Q kit), 0.077 µg/mg (P kit), 1.293 µg/mg, and 5.356 µg/mg, respectively (Table 1).

In case of RNA, the average concentration of extracted RNA was 70.58 ng/µl (BPC), 7.2 ng/µl (F&T) and 236.02 ng/µl (EMNE) (Table 2). The RNA yield of EMNE was 14.30 µg/mg, whereas the yield of BPC and F&T were 1.43 µg/mg and 0.1832 µg/mg, respectively.

### Purity of Extracted Nucleic Acids

The nucleic acids purity was estimated by calculating the ratio between the absorbance at 260 and 280 nm (*A_260_/A_280_*) and 260 and 230 nm (*A_260_/A_230_*). The *A_260_/A_280_* for the DNA prepared using the EMNE method was in the range of 1.72 to 2.05 and that of RNA was in the range of 1.72 to 1.94, indicating high quality nucleic acids for efficient downstream applications. The *A_260_/A_230_* for the DNA prepared using the EMNE method was in the range of 1.94 to 2.19 and that of RNA was in the range of 1.95 to 1.09, indicating little or no polysaccharide contamination, which is almost unavoidable in DNA extracted from *B. braunii*. Comparatively, *A_260_/A_230_* for DNA prepared using commercially kits was in the range of 0.23 to 0.58, which indicates high polysaccharide contamination (Table 1 and 2). DNA extracted from 1 ml and 3 ml broth of *B. braunii* using commercial kits resulted in DNA with low concentration and purity (data not shown).

### DNA Integrity Analysis

Real-time PCR experiments were performed to determine the integrity of DNA and cDNA obtained from each method. Absolute quantification of PCR amplification of different sizes of *rBcL* and 18 S rRNA genes revealed that the DNA and cDNA template obtained by EMNE method yielded higher PCR efficiency compared to other methods (Figure 4 and 5).

There was significant variance in the copy number values of 18 S rRNA genes and *rBcL* gene using both DNA and cDNA between the extraction methods (18 S rRNA gene from DNA, *P*-value <0.001; *rBcL* gene from DNA, *P*-value <0.05, 18 S rRNA gene from cDNA, *P*-value <0.001; *rBcL* gene from cDNA, *P*-value <0.001). This is primarily due to the fact that there is a significant increase in the gene copy number when EMNE is used when compared to other methods because of the quality and quantity of input DNA and RNA (Figure 4 and 5). Moreover, sample to sample variation is also high among all methods as reflected in very low P values.

### Cost Effectiveness Analysis

The cost effectiveness analysis involved calculation of costs of inputs as obtained from the manufacturer and effectiveness as obtained from the experimental results. A total of 5 parameters for determining effectiveness of each method, four parameters for costs and one parameter for time and labour were computed. The relative performance index method was used to rate the methods based on the results, with a baseline score of 10 indicating best performance (Table 3). EMNE method obtained a highest cost-effectiveness score of 72.09, followed by F&T and BPC at 60.17 and 58.45, respectively. P Kit performed better than Q Kit with a score of 53.22 and 46.23 respectively. The cost-effectiveness for RNA was not computed as costs for commercial kits is higher for RNA extraction as well as the gene quantification results obtained for EMNE method was equally promising using cDNA. Subsequently, cost effectiveness score for RNA extraction would have been similar or better for EMNE method.

### DNA Extraction from Other Green Algae and Diatom

EMNE method could be used to extract DNA from diatoms and green algae like *Botryococcus* sp. In this study, DNA was extracted from other microalgae (green algae; *C. reinhardtii* KCTC AG 20446, *Chlorella* sp. KCTC AG 10032, and *Scenedesmus* sp. KCTC AG 20831, diatom; *Phaeodactylum tricornutu* KCTC AG 30124 (10.26 mg) and *Pleurochrysis carterae* KCTC AG 40012) using EMNE method. The extracted total DNA from *C. reinhardtii*, *Chlorella* sp., *Scenedesmus* sp., *Phaeodactylum tricornutu* KCTC AG 30124, and *Pleurochrysis carterae* KCTC AG 40012 were 19.095 µg, 34.095 µg, 94.58 µg, 15.825 µg, and 12.975 µg, respectively. Corresponding, DNA yield was 11.787 µg/mg, 13.693 µg/mg, 17.041 µg/mg, 1.542 µg/mg, and 1.191 µg/ml, respectively (Table S1). Diatoms have a rigid cell wall as amorphous silica (i.e. glass) and silicified wall as organic coat [17]. EMNE is a very powerful method to extract the DNA from diatoms. The extracted DNA from 6 ml diatom broth culture showed excellent DNA extraction efficiency. However, EMNE method is not very sensitive for the extraction of DNA from cyanobacteria (data not shown).

## Discussion

A reliable method for nucleic acids extraction should meet the following criteria: (i) require only a small amount of sample (ii) involve simple procedures (iii) use minimal number and amount of chemicals (iv) yield high-quality and quantity of nucleic acids; and (v) should be cost-effective (vi) should be strain-independent [18].

The proposed EMNE method has all these advantages. In addition, EMNE method is also highly effective for downstream applications when compared to other methods. The real-time PCR results suggest that EMNE method not only yields high concentration of nucleic acids, but also yields nucleic acids with high integrity, ensuring both quality and quantity. RNA isolated using EMNE method is found to yield consistently better quantity and quality of RNA for all strains of *B. braunii* than most previously published methods.

The average DNA yield when using the EMNE method was about 300 times higher than that of commercial kit (P kit) (Table 1) and average RNA yield using EMNE method is about 10 times higher than BPC method. Moreover, EMNE method required fewer cell mass for extraction of DNA/RNA than other methods. DNA extracted from *B. braunii* using EMNE method can ensure minimum DNA yield of 9 µg/mg of cell and a minimum RNA yield of 14.20 µg/mg of cell with high purity. The EMNE method is also more cost-effective than commercial kits (>25% ) and previously published methods (>16%).

Statistical analysis of the qPCR data also indicate high quality and quantity of nucleic acids obtained from EMNE method. Co-variance data of copy number values from RT-qPCR for EMNE is very less (0.12) when compared to commercial kits, 0.17 and 0.42 for P Kit and Q Kit respectively. These results strongly highlight the effectiveness of EMNE method in obtaining high quality nucleic acid from recalcitrant microalgae.

Advantages of using EMNE method include,

- This is a simple, rapid and cost-effective method for isolation of DNA from eukaryotic microalgae.
- The method can also be used to isolate RNA from *B. braunii* with little difference in procedure and better cost-effectiveness.
- The method only requires small amount of sample, i.e. 1 ml, which is a boon for studies on recalcitrant microalgal strains.
- The method yields high quality nucleic acids which increases the efficiency of downstream applications.
- The method does not use any harmful chemicals which not only reduces direct costs, but also reduces indirect costs such as health costs and enhances indirect benefits.
- Finally, the method also requires far less time and effort compared to commercially available methods.

In conclusion, we declare that the EMNE is by far a superior method when compared to existing methods and commercial kits. To the best of our knowledge, this is the most simple and cost-effective method for co-isolation of DNA and RNA from a variety of eukaryotic microalgae.

## Supporting Information

Table S1 Comparison of quantity and quality of DNA obtained from different green algae using EMNE method.
(DOC)
