# Supplementary material for: Simple, Rapid and Cost-Effective Method for High Quality Nucleic Acids Extraction from Different Strains of Botryococcus braunii
Source: PLoS One. 2012 May 25;7(5):e37770. doi: 10.1371/journal.pone.0037770 (PMC3360624; doi:10.1371/journal.pone.0037770)
Supplement: Table S1 — Comparison of quantity and quality of DNA obtained from different green algae using EMNE method. (DOC) [file pone.0037770.s001.doc]

**Table S1**

| Taxonomic | A_260/280_^a^ | DNA conc. (ng/µl)^b^ | Total DNA (µg) | Dry Cell Weight | DNA yield (µg/mg)^c, d^ |
| --- | --- | --- | --- | --- | --- |
| *Phaeodactylum tricornutu* KCTC AG 30124 | 2.02 | 158.25 | 15.825 | 10.26 | 1.542 |
| *Pleurochrysis carterae* KCTC AG 40012 | 1.92 | 129.75 | 12.975 | 15.42 | 0.841 |
| *Chlamydomonas reinhardtii* KCTC AG 20446 | 1.92 | 190.95 | 19.095 | 1.62 | 11.787 |
| *Chlorella* sp. KCTC AG 10032 | 1.92 | 340.95 | 34.095 | 2.49 | 13.693 |
| *Scenedesmus* sp. KCTC AG 20831 | 2.07 | 945.8 | 94.58 | 5.55 | 17.041 |

^a^The DNA purity was estimated by calculating the ratio between the absorbance at 260nm and 280 nm.

^b^The DNA concentration was quantified using a spectrophotometer at 260 nm.

**^c^T**he cultured cells were harvested from culture of **6 ml (diatom) and 1 ml (EMNE method for green algae),** respectively.

^d^The DNA yield was estimated by calculating the DNA concentration between the dry cell weight and total DNA.

KCTC, Korean Collection for Type Cultures
